# Economic Viability of the Production of Peruvian Grunt (*Anisotremus scapularis*) in RAS on the Peruvian–Chilean Desert Coast

**DOI:** 10.3390/ani15010048

**Published:** 2024-12-28

**Authors:** Pablo Presa, Yolanda Leonor Perca Cruz, Jordan I. Huanacuni, Renzo Pepe-Victoriano, Luis A. Espinoza-Ramos

**Affiliations:** 1 Laboratory of Marine Genetic Resources (ReXenMar), CIM, Universidade de Vigo, 36310 Vigo, Spain; pressa@uvigo.gal; 2 Escuela de Ingeniería Pesquera, Universidad Nacional Jorge Basadre Grohmann, Tacna 23004, Peru; yolandaleonor.pc@hotmail.com (Y.L.P.C.); jordan.92ihp@gmail.com (J.I.H.); 3 Finfish Aquaculture, Sociedad Anónima Cerrada, Tacna 23004, Peru; 4 Facultad de Recursos Naturales Renovables, Área de Biología Marina y Acuicultura, Arica 1000000, Chile; rpepev@unap.cl; 5 Núcleo de Investigación Aplicada e Innovación en Ciencias Biológicas, Facultad de Recursos Naturales Renovables, Área de Biología Marina y Acuicultura, Universidad Arturo Prat, Iquique 1110939, Chile

**Keywords:** aquaculture diversification, economic viability, entrepreneurial transfer, marine aquaculture, Peruvian grunt

## Abstract

The Peruvian grunt *Anisotremus scapularis* is one of the most appreciated fish in Peruvian national markets. However, its reduced and irregular fishery is a paradigm of illegal, unreported, and unregulated fishing (IUU) in the Peruvian–Chilean coastal region. An important technological advancement has been achieved in the last decade in capture, management, nutrition, and broodstock maintenance to boost pilot experiences on the aquaculture of this species. Therefore, it is pertinent to evaluate the economic viability of this candidate species to identify cultivation bottlenecks, optimize the process, and assess the transfer feasibility of the technical know-how to interest groups. In this study, we performed a sensitivity analysis to assess how market price and production size should make its commercialization profitable. We show that the use of photovoltaic solar panels and a raw production cycle of 15 months enables profitability at a ~400 k unit farm size at a commercial market price of 6.67 USD/kg, i.e., B/C = USD 1.14. Both, time to market and market price exerted the highest influence on the value of grunt farms; whereas, a reduction of the production cycle to 12 months by seed selection, optimized rearing conditions, and nutritional diets also provides a profitable investment, i.e., NPV = USD 287,054 with an IRR = 23.71% at a discount rate of 10% and B/C = USD 1.15. These scenarios pave the way for the transfer of technological know-how to entrepreneurial initiatives in the economically depressed coastal region of the Atacama Desert.

## Simple Summary

Well-informed consumers demand world markets increase production transparence and adhere to sustainable fish husbandry practices. The Peruvian grunt inhabiting the rocky shores of American Southern Pacific has a high commercial value but its wild population is being harvested unsustainably. The cultivation of this grunt could alleviate fishing pressure on its natural population. Therefore, a bioeconomic insight would allow researchers to gauge cultivation bottlenecks and optimize processes as well as assess the transfer viability to the private sector. This study analyzes the economic feasibility of recirculating aquaculture systems (RAS) in Peruvian grunt farming using field data from previous technological developments. The sensitivity analysis shows that the economic outcome of this grunt aquaculture using photovoltaic solar panels and a 15-month production cycle results in a profitable investment for a farm size of about 400 K/yr. Likewise, a similar profitable exploitation can be achieved by shortening the production cycle to 12 months. These achievements would enable the know-how transference to entrepreneurial initiatives in the semi-desert region of southern Peru and northern Chile.

## 1. Introduction

The fishery sector has had a significant 1.3% contribution to the Peruvian GDP (gross domestic product) in the last decade [https://snp.org.pe/la-pesca-en-cifras/ (accessed on 14 July 2024)]. However, extractive fisheries exhibited a downward trend over the past decades mainly due to overfishing, pollution, and climate change [1]. Such stagnation of wild fisheries is fueling the development of aquaculture (growing systems and candidate species) [2], which has become the world’s fastest growing food production sector [3]. Nevertheless, pending environmental issues such as sustainable production practices and certification systems are still far from complete [4].

Growing interest exists in aquaculture in the semi-desert coastal region of southern Peru and northern Chile because of its economic and nutritional potential. The Peruvian Department of Tacna is located at the head of the Atacama Desert on the Pacific coast of South America. Its coastline extends from the “Concordia” line between Peru and Chile (18°21′08″ S, 70°22′39″ O) to the beaches of Punta Huaca Luna on its northern border with the Moquegua Department (17°11′33.8496″ S, 70°55′58.1304″ W). The Tacna coastal area is characterized by sandy shores and shallow waters [5] and, also, by more oxygenated and protective shores where a large variety of rocky fishes dwell [6,7]. While the highest landings for human consumption in Peru correspond to jack mackerel (*Trachurus murphyi*), tuna (*Sarda chiliensis chiliensis*), and “perico” (*Coryphaena hippurus*), some other species are in high demand in southern Peru, i.e., “cabrilla” (*Hemanthias peruanus*), the Peruvian croaker (*Cilus gilberti*), the Peruvian grunt (*Anisotremus scapularis*), the conger (*Genypterus maculatus*), and the swordfish (*Xiphias gladius*) [8].

The predominant economic activity on the coasts of Tacna is artisanal fishing with around 1040 fishermen (2.4% of the total national) with 34% share fishing on croaker (*Cilus gilberti*), lorna (*Sciaena deliciosa*), grunt (*A. scapularis*), mollusks (e.g., *Dosidicus gigas*, *Octopus mimus, Argopecten purpuratus*), crustaceans (*Cryphiops caementarius*), and echinoderms (*Patallus mollis*), for first sale in local markets and direct human consumption [5]. The processing and transformation sector of hydrobiological resources mainly exploit sea urchin eggs (*Loxechinus albus*), flying fish eggs (*Cypselurus heterurus*), and anchovy (*Engraulis ringens*) [8]. The annual consumption of hydrobiological products in the Tacna Region in 2021 (fresh, frozen, canned fish and shellfish) was 14.3 kg/capita [9], a ratio well below the national average of 17.3 kg/capita.

**Figure 1:**
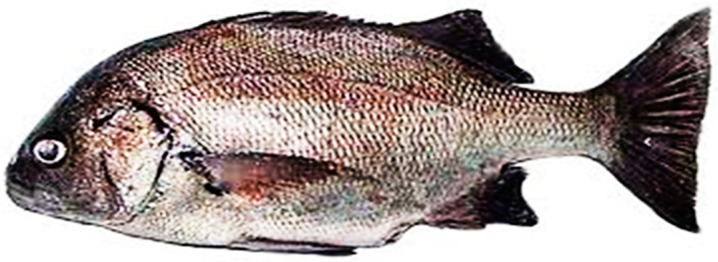
Adult specimen (60 cm in length) of the Peruvian grunt *Anisotremus scapularis*.

The Peruvian grunt *Anisotremus scapularis* is one of the most appreciated species in national markets and is locally known as “chita”, “sargo”, snorer “roncador”, or “corcovado” (Figure 1).

**Figure 2:**
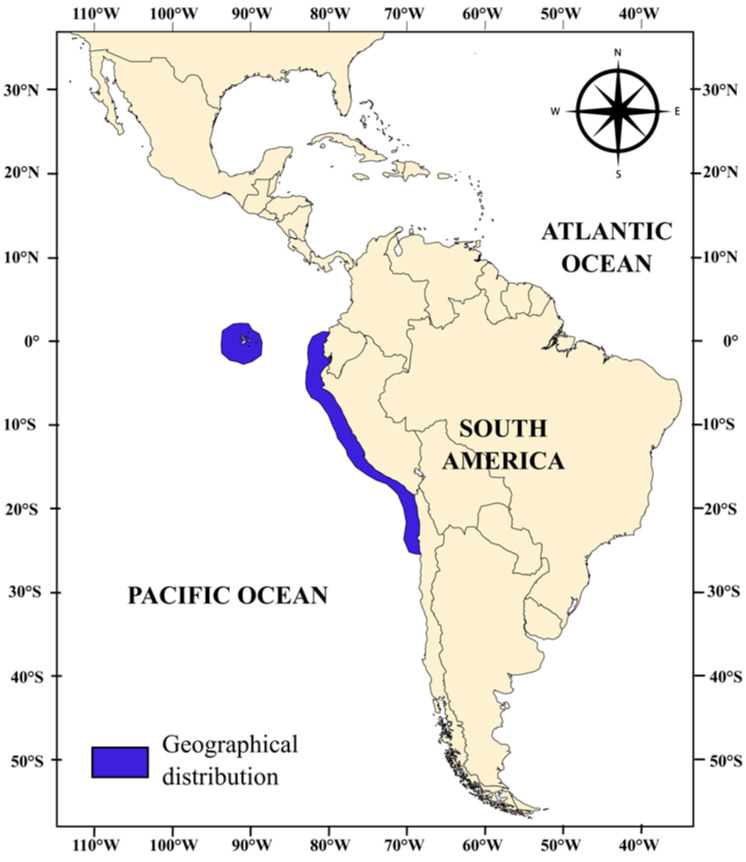
Distribution of *Anisotremus scapularis* [Tschudi, 1846, https://www.marinespecies.org/aphia.php?p=taxdetails&id=279622 (accessed on 5 August 2024)] in the Pacific south.

This grunt is one of the six species of the genus *Anisotremus* (*Actinopterygii*, *Perciformes*, *Haemulidae*) so far reported in Peruvian waters [10]. It is distributed along the coasts of Ecuador, Peru, and Chile, from Manta (Ecuador) to Antofagasta and Cocos Island (Chile) (Figure 2) in rocky areas down to 25 m in depth [11,12].

The adult grunt has a robust body, a convex head, and a small, low, and terminal mouth and reaches 80 cm in length and 7.5 kg in weight (Figure 1). Grunt juveniles are omnivorous, feeding on macroalgae and minor invertebrates, especially copepods, and aggregate in large schools in marine rocky shores and intertidal pools. Bentho-pelagic adults are lonelier and dwell on sandy bottoms; they euryphage on green and red algae as well as invertebrates such as amphipods, caprellids, ophiuroids, echiurids, polyplacophorous mollusks (e.g., *Chiton cumingsi*), and bivalves (e.g., *Semimytilus algosus*) [13].

The fishery of Peruvian grunt employs artisanal gears such as string, seine, curtain, and spinel. Landings of this grunt showed a high inter-annual variability, ranging 119–144 GMT (gross metric tons) in the period of 2017–2021. Those figures indicate that the Peruvian grunt fishery is one of the smallest and most irregular among Peruvian artisanal fisheries (Table 1). This shortage has affected the most vulnerable economic stratum made up of artisanal fishermen from small ports and coves in southern Peru. This subsector englobes seasonal coastal professionals and occasional low-expertise fishermen from the highlands with a very precarious economy [14]. The shortage of grunt due to overexploitation (not fully evaluated yet) has not only impacted the species’ abundance but also the consumer habits and the economic wealth of the tertiary sector [https://saludconlupa.com/medio-ambiente/pescadores-en-peru-enfrentan-a-los-ilegales-nos-hemos-organizado-para-trabajar-la-chita-pese-a-los-peligros/ (accessed on 7 August 2024)]. Altogether, the local scarcity of this species and its irregular commercialization in national markets make it a paradigm of illegal, unreported, and unregulated fishing (IUU) [15].

The grunt market value and its organoleptic excellence, together with the decline of its fishery, prompted a research project in October 2014 in southern Peru to develop the technological know-how for grunt aquaculture [16]. Eventually, the successful outcome of that initiative would boost the grunt local production as well as alleviate the harvesting pressure on its wild Pacific population. Thereafter, technologies for capture, management, nutrition, and broodstock maintenance were developed in the last decade [16,17,18,19,20].

In parallel to that zootechnical advancement, it is crucial to evaluate the economic viability of this grunt as an aquaculture candidate. Bioeconomic models are key tools for assessing the economic viability of aquaculture systems. These models allow for integrating biological factors such as growth and mortality rates with financial costs and market prices. In particular, horizontally integrated systems, which combine fish, mollusks, and macroalgae, have proven to be economically viable under certain scenarios and can help optimize productivity and reduce environmental impacts [21,22]. Most studies highlight the importance of operational costs and bioeconomic approaches, with differences between species requiring tailored approaches.

Therefore, the aim of this work is to perform a sensitivity analysis to assess the economic viability of Peruvian grunt farming in Vila—Vila, Tacna (Peru). Our null hypothesis considers that if simulated sea bream aquaculture is economically viable, then it is feasible to start a process of transferring the accumulated knowledge of its cultivation to potential entrepreneurs. In other words, this sensitivity analysis would allow us to gauge cultivation bottlenecks and optimizable processes to determine the economic potential of the incipient grunt aquaculture and its transfer viability to the private sector using escalated cultivation systems (e.g., sea cages) [23]. Notably, this area shares a common environmental setup with the rest of the desert coastal region of southern Peru and northern Chile where grunt aquaculture would find its natural niche.

## 2. Materials and Methods

Once the background knowledge on the cultivation of this species is achieved, the sensitivity analysis performed herein aims to portray the economic outcome of mass culturing the Peruvian grunt in a RAS system under different energy sources, farm sizes, and growth cycle spans. This research was carried out in 2022 at Escuela de Ingeniería Pesquera of University Jorge Basadre Grohmann (UNJBG, Tacna, Peru) using experimental data collected on this species since 2014 at the Morro Sama Aquaculture Center (CAMOSA) of the National Fisheries Development Fund (FONDEPES, Peru) (17°59′39.7″ S, 70°52′59.1″ W). All animal procedures and handling, as described in previous studies, e.g., [16], were carried out according to the Peruvian law#30407 «Law on Animal Protection and Welfare» governed by the Ministry of Agriculture and Irrigation (MINAGRI, Peru) [https://www.leyes.congreso.gob.pe/Documentos/Leyes/30407.pdf (accessed on 7 July 2024)].

### 2.1. Environmental Setup

**Figure 3:**
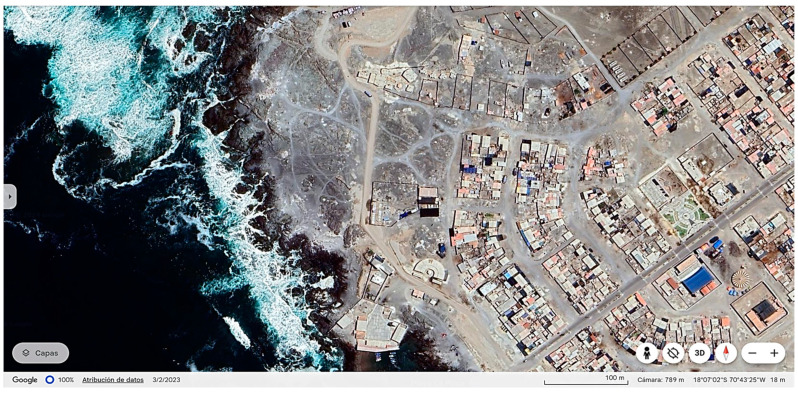
The environmental setup and location of the Coastal Marine Laboratory of Vila—Vila (Tacna, Peru) (Source: Coastal Marine Laboratory Satellite View [https://www.google.com/maps/place/Vila+Vila+Beach/@-18.1144445,-70.7278606,810m/data=!3m1!1e3!4m5!3m4!1s0x91453fd7f507c05b:0x6bf8ebfcbdca8f6c!8m2!3d-18.1172591!4d-70.7264604?hl=es-ES (accessed on 7 July 2024)].

The fishing village of Vila—Vila where the Coastal Marine Laboratory of UNJBG is located (Sama, Tacna, Peru) is the environmental setting with the hatchery facility employed to assess the economic viability of the incipient Peruvian grunt aquaculture (Figure 3). Given that the real area allocated for aquaculture exploitation is owned by farmers or by academia, its cost is not included in this economic assessment. Source data to calculate the initial investment, operation costs, and maintenance costs were funded by the UNJBG—CANON research contract “*Investigación y desarrollo de las tecnologías de cultivo de peces marinos de importancia económica: corvina (Cilus gilberti) y sargo (Anisotremus scapularis) en la región Tacna*” (Rectoral Resolution No. 3780-2014-UNJBG) and its scientific publications [16,17,18,19,20,24].

### 2.2. Structure of the Culture

The experimental facilities evaluated consisted of 3–20 fiberglass circular tanks implemented to maintain all life phases of the Peruvian grunt under controlled conditions. Those facilities include a RAS system with 100% water recirculation per hour and equipped with water quality controls for an optimal adaptation of the grunt broodstock, i.e., 18–19 °C temperature and 6.55 mg/L of dissolved oxygen [16]. Seawater inflow through pipelines had a multi-filtering system to guarantee water quality supply to culture tanks. The indoor facility evaluated was protected from direct sunlight in order to minimize evaporation and to maintain a cooler temperature than outside.

### 2.3. Biological Material

Peruvian grunt larvae as well as feedstuff were supplied by the Coastal Marine Laboratory of UNJBG according to the feeding protocols developed for the whole life cycle management of this species [19]. The feed conversion ratio (FCR) used in this simulation was 1.8; an FCR of 1.8 is a promising figure for a candidate species optimally fed since 2016. The initial culture of grunt larvae was enforced at an average density of 30 larvae/L. Grunt fry used in current calculations averaged 3.80 ± 0.31 cm in fork length and 0.53 ± 0.22 g in live weight and were cultured in seven tanks of 47 m^3^ of volume (Table S1). Grunt juveniles 1+ in those tanks ranged 5–10 cm in fork length and averaged 20.00 ± 0.09 g in live weight; their culture averaged 10 kg/m^3^ (470 kg/tank) with density ranging from 0.6 kg/m^3^ (282 kg/tank) at day 1 of cultivation to 15 kg/m^3^ (705 kg/tank) at day 75 of cultivation (Table S1). Grunt juveniles 2+ averaged 15.46 ± 1.01 cm in fork length (range 12–16 cm) and 160.00 ± 35.01 g in live weight; their culture was enforced at an average density of 20 kg/m^3^ in fifteen tanks of 81 m^3^ of volume (Table S2). The commercial size of post-fattening adult grunts was >20 cm in fork length with an average live weight of 300.00 ± 65.62 g; their culture density was enforced at 26 kg/m^3^ in twenty circular tanks of 84 m^3^ of volume (Table S3).

### 2.4. Sensitivity Analysis

The production output of aquacultured organisms is constrained by costs incurred from the initial investment, the management practices, and the production process [2]. Capital budgeting is an appropriate approach for assessing the economic feasibility of the current Peruvian grunt farming system. For this species, a sensitivity analysis was developed herein to assess the weight of variable inputs on the final benefit-to-cost ratio (B/C). The initial investment costs included civil work facilities, administrative equipment, cultivation equipment, and machinery (Table S4). Variable costs comprised tank preparation before stocking (salt, fungicide, etc.), fingerling, feed, energy, repair and maintenance, sludge discharge, veterinary services, and labor (Table S5). The working capital was calculated on a 15-month production cycle (Table S6) following the official labor costs of the country. Fixed costs consisted of depreciation of equipment and replacement costs whenever the useful life of the equipment was shorter than the evaluation horizon (Table S7). Total production costs and revenues were expressed in USD (applied exchange rate: USD 1.00 equaled PEN 3.75 as of 1 December 2024) (Table S8). The final product was a Peruvian grunt of ~300 g of live weight (according to customers’ preferred serving size) after a 15-month production cycle and the commercial scenario comprised local fish markets of the Tacna Department (Table S9).

The indicators applied to the economic evaluation were the net present value (NPV), i.e., the difference between the present value of cash inflows and the present value of cash outflows over 10 years; the internal rate of return (IRR), i.e., the return estimated from the investment; and the benefit–cost ratio (B/C), i.e., a profitability index that measures the projected capital inflow (benefits) on the investment (costs). The NPV was determined as the sum of funds flow as updated at a previously calculated discount rate, using the mathematical formula

$$NPV=-I_{0}+\sum_{t=1}^{n}\frac{F_{n}}{{\left(1+i\right)}^{t}}$$

where *I*_0_ is the initial investment; *F_n_* is the net flow in period *t*; *t*, is the project horizon (in yr); *i*, is the discount rate applied [25].

Profitability ratios are financial calculations to assess profitability upon costs and earnings for a specific time period [26]. The profitability ratios employed herein were the internal rate of return (IRR) and the benefit–cost ratio (B/C). The internal rate of return (IRR) is the annual rate of growth at which the updated value of the net funds flow (NPV) equals zero, and it was calculated as

$$IRR=-I_{0}+\sum_{t=0}^{n}\frac{F_{n}}{{\left(1+i\right)}^{t}}=0$$

The benefit–cost ratio (B/C) as a profitability indicator that summarizes the overall value for money of this project was calculated as the result of dividing the updated value of the benefit stream by the updated value of the cost stream at a previously determined update rate. B/C < 1 indicates loss, a B/C = 1 indicates break-even, and a B/C > 1 indicates profit [27]. Herein, B/C was calculated as

$$B/C=\frac{\sum_{t=1}^{n}\frac{B_{n}}{{\left(1+i\right)}^{t}}}{\sum_{t=1}^{n}\frac{C_{n}}{{\left(1+i\right)}^{t}}}$$

where *B_n_* is the net profit in period *t*, and *C_n_* is the net cost in period *t*.

## 3. Results and Discussion

Diversification in aquaculture is relevant for coastal communities experiencing the backlash of declining wild fisheries [https://ec.europa.eu/archives/commission_2010-2014/damanaki/headlines/press-releases/2012/09/20120913_en.htm (accessed on 17 May 2024)]. Most studies in this field agree that the profitability of aquaculture candidates depends significantly on local factors, such as labor costs, environmental regulations, access to markets, and a commercial value of the resource [28,29]. The Peruvian grunt (*Anisotremus scapularis*) is a species of growing interest in marine aquaculture due to a high demand for it in local markets and its ability to adapt to farming conditions. However, the economic evaluation of its farming still faces challenges related to production costs, integration into sustainable systems, and access to competitive markets.

The sensitivity analysis performed herein on the potentiality of Peruvian grunt aquaculture indicates the break-even point of grunt production (B/C = 1) at 397,540 grunt units. In terms of production, this implies that a ~400 k unit farm size at a commercial market price of 6.67 USD/kg provides profitable exploitation (B/C = USD 1.14). Nevertheless, a similar outcome can be achieved by shortening the productive life cycle to 12 months, i.e., NPV = USD 287,054 with an IRR = 23.71% at a discount rate of 10% and B/C = USD 1.15.

### 3.1. Initial Investment

The initial investment included the acquisition of tangible or intangible assets as necessary to initiate the cultivation system. In the calculation with photovoltaic solar panels, the fixed investment amounted to USD 234,376 (Table S4). The working capital amounted to USD 162,765 (Table S6) as calculated for 15 months until the grunt production was commercialized and is now linked to the estimated production of the grunt. This capital allowed for daily operations and current expenses to be covered without cash flow shortage in the short term, thereby guaranteeing food purchase and operating expenses in the face of unexpected situations.

### 3.2. Costs

In order to evaluate the economic profitability, the evaluation horizon comprised a life period of 10 years for the main assets. These costs amounted to USD 54,896 (Table 2).

The variable costs were mainly caused by the purchase of larvae, the feeding costs, and the occasional electrical supply to complement the photovoltaic energy. Therefore, these costs depend on production, i.e., on the grunt culture escalation (Table S5). Similar findings have been reported from tilapia production in Lake Victoria [30] as well as in a study of cost drivers in US aquaculture where the cost of feed and fish seed were among the highest in all aquaculture production systems [31]. Also, horizontally integrated systems have been evaluated in temperate and warm waters, highlighting that efficient nutrient and resource management is essential to increasing economic returns [28]. This pattern is repeated in intensive and semi-intensive aquaculture systems, regardless of the geographic region. Notably, feed and energy costs stand out as the main factors affecting profitability in marine aquaculture systems. These costs represent up to 60% of total operating expenses in integrated systems [28], which could also apply to Peruvian grunt farming given that its diet requires high-quality feed to ensure optimal growth and quality of the final product.

The production costs encompass all the resources and activities required during the production process and refer to the set of expenses and disbursements necessary to produce this grunt or to provide services. They include the fixed costs and the variable costs, which altogether amounted to USD 111,754 in year 1 and SD 98,966 in year 10 (Table S8). The depreciation of fixed assets is defined as the loss of their value over their useful lifespan. Those assets depreciating before the ten-year horizon were replaced one year after the loss of their useful life (Table S7).

**Figure 4:**
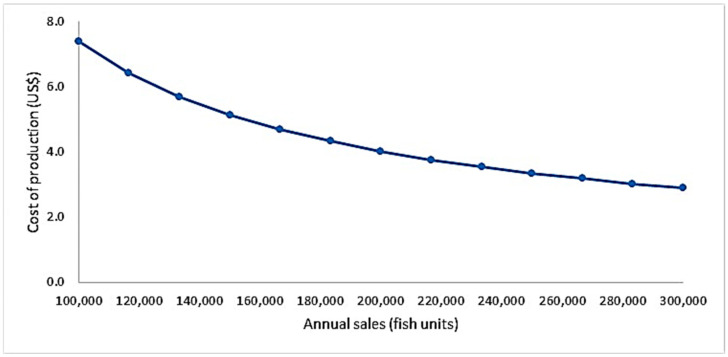
Aggregated cost of production per grunt unit as a function of farm size (annual sales).

A progressive reduction in production costs was observed with the increase in the production scale, i.e., current simulations indicate that annual sales >300,000 grunt units reduced production costs below 3 USD/unit (Figure 4).

### 3.3. Economic Indicators

In order to establish the cash flow in the evaluation horizon, we first present the detail of the income flow from the sale of the grunt with a commercial weight of 300 g/specimen and a 10% natural mortality (Table S9). Thereafter, we show the cash flow employed to estimate the NPV (Table S10). A negative net present value (NPV) in USD was obtained upon the cash flow expected to be generated during the useful life of the project in both situations, i.e., without photovoltaic solar panels (NPV = −USD 258,716) and with them (NPV = −USD 86,758) (Table 3). Likewise, the internal rate of return (IRR) of −3% was obtained without photovoltaic solar panels, which suggests that grunt production with electrical supply would not generate enough income to achieve an acceptable profit at the discount rate applied. The use of photovoltaic solar panels improved the situation significantly with the IRR becoming positive (7%) despite the NPV still being negative.

The benefit–cost ratio (B/C) as a measure that allows for comparing the benefit expected with the costs incurred, indicates the absence of profit under both situations, i.e., either without solar panels (USD 0.25) or with them (USD 0.95), with neither of them reaching the expected profitability in relation to the opportunity cost (Table 3).

**Figure 5:**
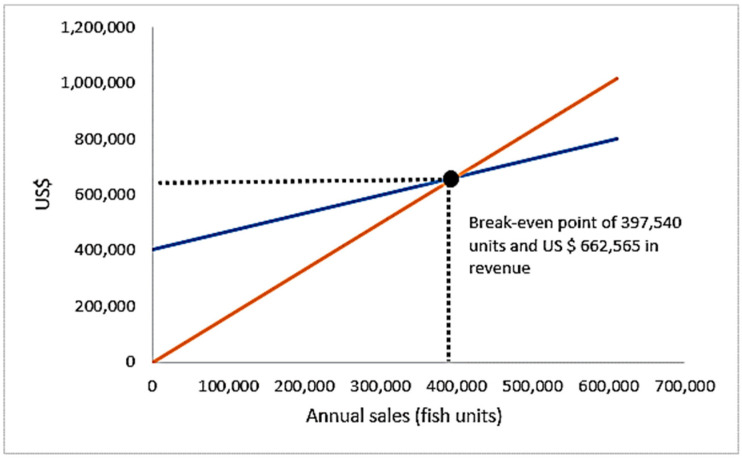
Sensitivity analysis indicating the break-even point of grunt production (B/C = 1) at 397,540 grunt units.

**Figure 6:**
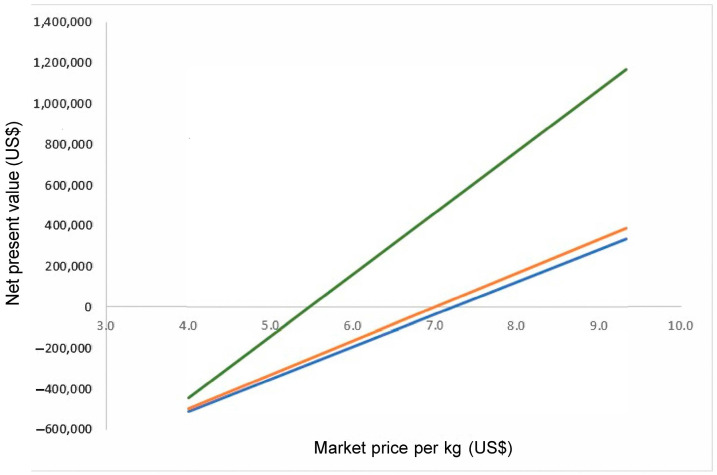
Net present value (USD; 10-year horizon) per market price (USD/kg) for a farm size of 160 K (blue), 200 K (orange), and 400 K (green) grunt units.

The sensitivity analysis indicates the break-even point of grunt production (B/C = 1) at 397,540 grunt units (Figure 5). In terms of production, this implies that a ~400 k unit farm size at a commercial market price of 6.67 USD/kg provides a clearly profitable exploitation (B/C = USD 1.14) (Figure 6).

The initial proposal to cultivate the Peruvian grunt without photovoltaic solar panels assumed a production cycle of 15 months after the cultivation protocol was designed for the Peruvian croaker Cilus gilberti [32]. However, as indicated above, the economic indicators of profitability were all negative, even with the implementation of photovoltaic solar panels. Under the latter, a minimum farm size threshold of ~400 k units provides clear profitability which can also be achieved by shortening the production cycle. This can be achieved by selecting high-growth rate seeds, e.g., as shown in mussels [33], environmental optimization, and balanced nutritional diets for all life stages. For instance, the reduction of the production cycle to 12 months would result in a profitable investment, i.e., NPV = USD 287,054 with an IRR = 23.71% at a discount rate of 10% and B/C = USD 1.15.

Some previous sensitivity studies prioritize long-term environmental sustainability by incorporating social and environmental variables, while others focus on maximizing immediate financial returns [29,34]. For instance, the systems evaluated in Israel and France [28] showed significant differences in initial investment and expected returns depending on the emphasis on sustainability or short-term performance. Strategies range from reducing initial costs through vertical integration to diversifying into premium products, such as organic foods. In the case of sea bream, these strategies need to be combined to address both market challenges and production costs.

## 4. Conclusions

The effective transition from overharvested wild fisheries to aquaculture requires explicit benefits to local communities and limited disruption of existing social patterns [35]. The current pioneering assessment of the economic profitability of the cultivation of the Peruvian grunt has been carried out on real data from an open circulation culture system, employing larvae supply from an experimental pilot laboratory as well as live and concentrated food supply. There are opportunities to reduce production costs, such as using alternate offshore wind energy coupled with marine cage aquaculture. The sensitivity analysis aimed to analyze how much the farm size should be increased to make grunt production profitable. Based on current information on costs and benefits, inshore open system production of Peruvian grunt using solar panels and a 15-month production cycle is profitable above a critical farm size of 400 K/yr. Time to market and market price also had a great influence on the value of grunt farms. Therefore, focusing efforts on optimizing grunt growth rates and developing markets for the added value of farmed grunt products could substantially increase farm values. This scenario paves the way for the transfer of technological know-how to new entrepreneurs in the economically depressed coastal region of the Atacama Desert.

## Figures and Tables

**Table 1:** Landings of main rocky coast fishes from Peru in the period of 2017–2021 (in gross metric tons).

Species (Common Name)	2017	2018	2019	2020	2021
*Isacia conceptionis* (Cabinza grunt)	1797	1934	1842	1137	1638
*Seriolella violacea* (Palm ruff)	255	158	234	269	112
*Cilus gilberti* (Peruvian croaker)	1501	1394	757	836	424
*Anisotremus scapularis* (Peruvian grunt)	119	171	141	179	144
*Mugil cephalus* (Flathead grey mullet)	32,275	25,190	25,985	16,151	16,053
*Sciaena deliciosa* (Lorna rum)	10,879	7024	3306	3096	1461
*Ethmidium maculatum* (Pacific menhaden)	2785	1318	2585	5450	2345
*Odontesthes regia* (Peruvian silverside)	3267	2956	4244	6336	5201
*Cheilodactylus variegatus* (Peruvian morwong)	156	229	295	301	254
Total landings	53,034	40,374	39,390	33,754	27,631

Source: Ministerio de la Producción—Oficina General de Evaluación de Impacto y Estudios Económicos [8].

**Table 2:** Fixed costs upon average Peruvian labor costs, which are invariant with production and were afforded irrespective of the volume of products, services, or the income generation.

Item	Description	Wages USD/Month	Annual Cost USD
Staff			45,120
	Administrator	933	11,200
	Aquaculture Engineer	853	10,240
	Operator 1	587	7040
	Operator 2	587	7040
	Guardianship 1	400	4800
	Guardianship 2	400	4800
Services			976
	Water ^1^	24	288
	Internet and telephone	33	400
	Various supplies	24	288
Maintenance			4000
	Equipment		2667
	Facility		1333
Displacements	Car rental		4800
Total			54,896

^1^ Water consumption for system replacement upon evaporation.

**Table 3:** Internal rate of return with or without photovoltaic solar panels.

	Without Photovoltaic Solar Panels	With Photovoltaic Solar Panels	400 K Units Production at 6.67 USD/kg ^1^
Opportunity cost	10%	10%	10%
NPV	−USD 258,716	−USD 86,758	USD 364,495
IRR	−3%	7%	20.53%
B/C	USD 0.23	USD 0.95	USD 1.14

^1^ The ex-factory price of 6.67 USD/kg ensures that the economic analysis focuses exclusively on production costs. This price is considered reasonable as it is located within an intermediate range between species of popular consumption in local markets such as horse mackerel (Trachurus murphyi), mackerel (Scomber scombrus) (2–3 USD/kg), and cojinova (Seriolella violacea) (7–10 USD/kg), with this latter species sharing similar market characteristics to the Peruvian grunt in terms of demand and commercial profile.

## Data Availability

Data are provided either within the article or in Supplementary Materials. Further technical data can be accessed upon request made to the corresponding author.

## Supplementary Materials

The following supporting information can be downloaded at: https://www.mdpi.com/article/10.3390/ani15010048/s1, Table S1: Characteristics of cultivation tanks for Peruvian grunt fry and juveniles 1+; Table S2: Characteristics of cultivation tanks for Peruvian grunt juveniles 2+; Table S3: Characteristics of cultivation tanks for Peruvian grunt adults (fattening period); Table S4: Broken-down initial investment (USD) on Peruvian grunt production as computed on photovoltaic solar panels; Table S5: Variable costs (USD) for the Peruvian grunt calculated on a 10-year horizon; Table S6: Working capital (USD) for a 15-month production cycle of Peruvian grunt in a RAS system; Table S7: Depreciation of fixed assets (USD) with replacement one year after the loss of their useful life; Table S8: Total production costs (USD) comprising fixed costs and variable costs as calculated on a 10-year horizon; Table S9: Grunt sales and income flow (USD) for a specimen weight of 300 g/unit at 15 months and 10% mortality, of a commercial production of 43 K for local fish markets; Table S10: Net cash flow (USD) on a 10-year evaluation horizon implementing photovoltaic solar panels as power supply.
