# Supplementary material for: Economic Viability of the Production of Peruvian Grunt (Anisotremus scapularis) in RAS on the Peruvian–Chilean Desert Coast
Source: Animals (Basel). 2024 Dec 28;15(1):48. doi: 10.3390/ani15010048 (PMC11718966; doi:10.3390/ani15010048)
Supplement: Supplementary file 1 [file animals-15-00048-s001.zip › animals-3287815-supplementary.pdf]

Economic viability of the production of Peruvian grunt (*Anisotremus scapularis*) in RAS on the Peruvian-Chilean desert coast

Pablo Presa[1], Yolanda Leonor Perca Cruz[2], Jordan I. Huanacuni[2,3], Renzo Pepe-Victoriano[4], Luis Espinoza-Ramos[2,*]

SUPPLEMENTARY MATERIAL

Table S1. Characteristics of cultivation tanks for Peruvian grunt fry and juveniles 1+.

Year	Fry and juveniles 1+	No. individuals	Maximum Individual weight (gr)	Total weight (kg)	Density (kg/m^3)	Water volume (m^3)	Tank volume (m^3)	No. required tanks	No. tanks enforced
1	80,000	80,000	20	1,600	10	200	47	3.44	3
2	160,000	160,000	20	3,200	10	220	47	6.88	7
3	160,000	160,000	20	3,200	10	280	47	6.88	7
4	160,000	160,000	20	3,200	10	280	47	6.88	7
5	160,000	160,000	20	3,200	10	280	47	6.88	7
6	160,000	160,000	20	3,200	10	280	47	6.88	7
7	160,000	160,000	20	3,200	10	280	47	6.88	7
8	160,000	160,000	20	3,200	10	280	47	6.88	7
9	160,000	160,000	20	3,200	10	280	47	6.88	7
10	160,000	160,000	20	3,200	10	280	47	6.88	7

Table S2. Characteristics of cultivation tanks for Peruvian grunt juveniles 2+.

Year	Juveniles 1+/2+	No. individuals	Maximum Individual weight (gr)	Total weight (kg)	Density (kg/m^3)	Water volume (m^3)	Tank volume (m^3)	No. required tanks	No. tanks enforced
1	76,000	76,000	160	12,160	20	608	81	7.51	8
2	152,000	152,000	160	24,320	20	1216	81	15.01	15
3	152,000	152,000	160	24,320	20	1216	81	15.01	15
4	152,000	152,000	160	24,320	20	1216	81	15.01	15
5	152,000	152,000	160	24,320	20	1216	81	15.01	15
6	152,000	152,000	160	24,320	20	1216	81	15.01	15
7	152,000	152,000	160	24,320	20	1216	81	15.01	15
8	152,000	152,000	160	24,320	20	1216	81	15.01	15
9	152,000	152,000	160	24,320	20	1216	81	15.01	15
10	152,000	152,000	160	24,320	20	1216	81	15.01	15

Table S3. Characteristics of cultivation tanks for Peruvian grunt adults (fattening period).

Year	Adults	No. individuals	Maximum Individual weight (gr)	Total weight (kg)	Density (kg/m^3)	Water volume (m^3)	Tank volume (m^3)	No. required tanks	No. tanks enforced
1	72,200	72,200	300	21,660	26	833	84	9.92	10
2	144,400	144,400	300	43,320	26	1,666	84	19.84	20
3	144,400	144,400	300	43,320	26	1,666	84	19.84	20
4	144,400	144,400	300	43,320	26	1,666	84	19.84	20
5	144,400	144,400	300	43,320	26	1,666	84	19.84	20
6	144,400	144,400	300	43,320	26	1,666	84	19.84	20
7	144,400	144,400	300	43,320	26	1,666	84	19.84	20
8	144,400	144,400	300	43,320	26	1,666	84	19.84	20
9	144,400	144,400	300	43,320	26	1,666	84	19.84	20
10	144,400	144,400	300	43,320	26	1,666	84	19.84	20

Table S4. Broken down **i**nitial investment (US$) on Peruvian grunt production as computed on photovoltaic solar panels.

Item	Description	Measurement	Amount	Unit price	Total
Civil works					80,000
	Installation of office module and environments	unit	1	80,000	80,000
Cultivation equipment and machinery					151,629
	Fry tanks	unit	7	744	5,208
	Juvenile tanks	unit	15	4,000	60,000
	Fattening tanks	unit	10	4,000	40,000
	Centrifugal pump 1.5 HP	unit	1	403	667
	Centrifugal pump 2 HP	unit	4	667	1,611
	Centrifugal pump 10 HP	unit	6	693	4,160
	Aerator pump 2.5 HP	unit	7	1,023	7,159
	Air and water system	unit	1	1,079	1,079
	Solar panel kit	Kit	1	8,533	8,533
	Electrical network installation	service	1	400	400
	Mechanical filters	unit	3	4,480	13,440
	Electric generator	unit	1	1,781	1,781
	UV filters	unit	5	360	1,800
	Manual pallet truck	unit	1	1,725	1,725
	Vane aerator	unit	1	160	160
	Balance	unit	1	93	93
	Buckets 10L	unit	25	5	133
	Harvest boxes	unit	120	11	1,280
	Tubs	unit	10	27	267
	Buckets 20L	unit	10	5	53
	Electronic scale 40kg	unit	2	40	80
	Oxymeter	unit	1	1,200	1,200
	Freezer	unit	1	800	800
Administrative equipment					2,747
	Melamine desk & shelf	unit	1	200	200
	Melamine management desk	unit	1	267	267
	Swivel chair	unit	1	67	67
	Computer	unit	1	933	933
	Melamine File Cabinet Shelf	unit	2	133	267
	Chairs	unit	6	13	80
	Multifunctional printer	unit	2	200	400
	Miscellaneous software	kit	1	533	533
Total					234,376

Table S5. Variable costs (US$) for the Peruvian grunt calculated on a 10-year horizon.

Item		1	2	3	4	5	6	7	8	9	10
Fry		12,800	25,600	25,600	25,600	25,600	25,600	25,600	25,600	25,600	0
	Amount (US$)	80,000	160,000	160,000	160,000	160,000	160,000	160,000	160,000	160,000	0
	Cost (US$)[1]	0.16	0.16	0.16	0.16	0.16	0.16	0.16	0.16	0.16	0.16
Feeding		44,352	86,688	86,688	86,688	86,688	86,688	86,688	86,688	86,688	44,352
	Annual production in tons	22	43	43	43	43	43	43	43	43	22
	Feed conversion factor FCR	1.80	1.80	1.80	1.80	1.80	1.80	1.80	1.80	1.80	1.80
	Cost per ton of pellets (US$)[2]	1,120	1,120	1,120	1,120	1,120	1,120	1,120	1,120	1,120	1,120
Electrical energy cost		564	564	564	564	564	564	564	564	564	564
	Average monthly consumption (kW)	350	350	350	350	350	350	350	350	350	350
	Average monthly cost (US$)	47	47	47	47	47	47	47	47	47	47
Total		57,716	112,852	112,852	112,852	112,852	112,852	112,852	112,852	112,852	44,916

[1]Cost to produce a grunt fry as estimated on a local in-factory-maintained productive broodstock [20].

[2]Current ex-factory cost of feedstuff for the Peruvian grunt upon a 15-month growth cycle.

Table S6. Working capital (US$) for a 15-month production cycle of Peruvian grunt in a RAS system.

Ítem	1	2	3	4	5	6	7	8	9	10	11	12	13	14	15
Costs															
Fixed costs	4,575	4,575	4,575	4,575	4,575	4,575	4,575	4,575	4,575	4,575	4,575	4,575	4,575	4,575	4,575
Variable costs	1,137	1,706	2,843	4,549	4,549	5,117	5,686	6,254	6,254	6,254	6,254	6,254	12,429	12,429	12,429
Total Costs	5,712	6,281	7,418	9,123	9,123	9,692	10,261	10,829	10,829	10,829	10,829	10,829	17,004	17,004	17,004
Total working capital															162,765

Table S7. Depreciation of fixed assets (US$) with replacement one year after the loss of their useful life.

Description	Measurement	Quantity	Unit cost	Total cost	Useful life (yr)	Residual value	Annual installment value
Fry tanks	unit	7	744	5,208	10	1,563	365
Tanks for juveniles	unit	15	2,667	40,000	10	12,001	2,800
Tanks for fattening	unit	10	4,000	40,000	10	12,001	2,800
1.5 HP centrifugal pump	unit	1	403	403	8	200	58
2 HP centrifugal pump	unit	4	667	1,611	8	483	141
10 HP centrifugal pump	unit	6	693	4,160	8	1,248	364
Aerator pump	unit	8	1,023	8,181	7	2,455	716
Air and water system	unit	1	1,079	1,079	15	324	50
Mechanical filter	unit	3	4,480	13,440	8	4,033	1,176
Electric generator	unit	1	1,781	1,781	6	534	208
Electrical resistance system	unit	5	1,672	8,360	8	2,508	731
Solar panel kit	Kit	1	8,533	8,533	25	4,000	1,167
UV filters	unit	5	360	1,800	8	540	158
Manual pallet truck	unit	1	1,725	1,725	8	518	151
Vane aerator	unit	1	160	160	8	48	14
Balance	unit	1	93	93	9	28	7
Buckets 10L	unit	25	5	133	6	40	15
Harvest boxes	unit	120	11	1,280	6	384	149
Tubs	unit	10	27	267	6	80	31
Buckets 20L	unit	10	5	53	6	16	6
Electronic scale 40kg	unit	2	40	80	9	24	6
Oxymeter	unit	1	1,200	1,200	6	360	140
Freezer	unit	1	800	800	6	240	93
Melamine desk & shelf	unit	1	200	200	6	60	23
Melamine management desk	unit	1	267	267	6	80	31
Swivel chair	unit	1	67	67	6	20	8
Computer	unit	1	933	933	6	280	109
Melamine file cabinet shelf	unit	2	133	267	6	80	31
Chairs	unit	6	13	80	6	24	9
Multifunctional printer	unit	2	200	400	6	120	47
					Total	44,292	11,607

Table S8. Total production costs (US$) comprising fixed costs and variable costs as calculated on a 10-year horizon.

Year	Fixed costs	Variable costs	Operating cost
1	54,896	57,716	111,754
2	54,896	112,852	167,887
3	54,896	112,852	167,887
4	54,896	112,852	167,887
5	54,896	112,852	167,887
6	54,896	112,852	167,887
7	54,896	112,852	167,887
8	54,896	112,852	167,887
9	54,896	112,852	167,887
10	54,896	44,916	99,812
Total	548,960	1,004,856	1.554.662

Table S9. Grunt sales and income flow (US$) for a specimen weight of 300 g/unit at 15 months and 10% mortality for a commercial production of 43K in local fish markets.

Year	1	2	3	4	5	6	7	8	9	10
Annual production (kg)[1]	-	43,200	43,200	43,200	43,200	43,200	43,200	43,200	43,200	43,000
Price (US$)[2]	-	6.67	6.67	6.67	6.67	6.67	6.67	6.67	6.67	6.67
Total (US$)	-	288,000	288,000	288,000	288,000	288,000	288,000	288,000	288,000	286,667

[1] As estimated from the Von Bertalanffy growth curve and the average individual specific growth rate of juveniles and adults of this species [20].

[2] The ex-factory price of 6.67 USD/kg ensures that the economic analysis focuses exclusively on production costs. This price is considered reasonable as it is located within an intermediate range between species of popular consumption in local markets such as horse mackerel (*Trachurus murphyi*), mackerel (*Scomber scombrus*) (2 - 3 US$/kg) and cojinova (*Seriolella violacea*) (7 - 10 US$/kg), this latter a species that shares similar market characteristics to the Peruvian grunt in terms of demand and commercial profile.

Table S10. Net cash flow (US$) on a 10-year evaluation horizon implementing photovoltaic solar panels as power supply.

	0	1	2	3	4	5	6	7	8	9	10
Investment	397,141							7,728		40,104	
Civil works	80,000										
Cultivation equipment and machinery	151,629										
Administrative equipment	2,747										
Replacement investments								7,728		40,104	
Working capital	162,765										
Income	0		288,000	288,000	288,000	288,000	288,000	288,000	288,000	288,000	286,667
Grunt sales			288,000	288,000	288,000	288,000	288,000	288,000	288,000	288,000	286,667
Costs	0	179,104	179,104	179,104	179,104	179,104	179,104	179,104	179,104	179,104	154,864
Fixed costs		54,896	54,896	54,896	54,896	54,896	54,896	54,896	54,896	54,896	54,896
Variable costs		112,601	112,601	112,601	112,601	112,601	112,601	112,601	112,601	112,601	44,070
Depreciation		11,607	11,607	11,607	11,607	11,607	11,607	11,607	11,607	11,607	11,607
Book value											44,292
Profit before taxes	-397,141	-179,104	108,896	108,896	108,896	108,896	108,896	101,168	108,896	68,792	131,802
Income tax	0	0	32,124	32,124	32,124	32,124	32,124	29,845	32,124	20,294	38,882
Profit after taxes	-397,141	-179,104	76,772	76,772	76,772	76,772	76,772	71,323	76,772	48,498	92,921
Working capital recovery											162,765
Book value											44,292
Net cash flow[1]	-397,141	-179,104	76,772	76,772	76,772	76,772	76,772	71,323	76,772	48,498	299,977

[1] Depreciation h**as** not b**een** included in the cash flow, therefore the benefit that could improve the economic viability of the project is omitted.
